# Replication of TCF7L2 rs7903146 association with type 2 diabetes in an Iranian population

**DOI:** 10.1590/S1415-47572010005000056

**Published:** 2010-09-01

**Authors:** Mahsa M. Amoli, Parvin Amiri, Javad Tavakkoly-Bazzaz, Elham Charmchi, Jila Hafeziyeh, Mohammad Keramatipour, Maryam Abiri, Shirin Hasani Ranjbar, Bagher Larijani

**Affiliations:** 1 Endocrinology and Metabolism Research Centre, Tehran University of Medical Sciences, TehranIran; 2 Department of Medical Genetics, Tehran University of Medical Sciences, TehranIran

**Keywords:** TCF7L2, gene, polymorphism, T2DM

## Abstract

The transcription factor 7-like 2 gene (TCF7L2) rs7903146 T allele is constantly associated with Type 2 diabetes in various populations and ethnic groups. Nevertheless, this has not been observed in two studies involving Arab populations. The aim of the present study was to investigate the association between TCF7L2 rs7903146 in an Iranian population. Type 2 diabetes patients (N = 258) and normal healthy control subjects (N = 168) from the same area, were examined. The ARMS- PCR (Amplification Refractory Mutation System) technique, subsequently validated by direct sequencing, was used for genotyping. Allele and genotype frequencies were significantly different between patients and controls TT *vs.* CT + CC [p 0.0081 OR 3.4 95%CI (1.27-11.9)] and T *vs.* C allele [p 0.02 OR 1.4 95%CI (1.03-1.9)]. Our data thus confirm the association between the rs7903146 T allele and T2D in an Iranian population, contrary to previous reports in Arab populations. This can possibly be attributed to differences in ethnic background or the effects of environmental factors.

Type 2 diabetes (T2D) is a common complex disorder resulting from the interplay of genetic and environmental factors (Gerich, 1998; Barroso, 2005). Following the report of a strong association between type 2 diabetes and a microsatellite marker located within intron 3 of the transcription factor 7-like 2 gene (TCF7L2) locus (Grant *et al.*, 2006), a single nucleotide polymorphism (SNP) (rs7903146 allele T) within this gene was found to be in linkage disequilibrium with the said marker. This variant has been consistently associated with type 2 diabetes (T2D) in European, Asian and African populations (Cauchi *et al.*, 2007), thereafter confirmed by gene-locus genotyping (Cauchi *et al.*, 2007).

Other genetic loci, including PPARG and KCNJ11, have also been reproducibly associated with type 2 diabetes. More recently, 16 novel T2D susceptible loci have been identified (Sparsø *et al.*, 2009). However, the magnitude of the TCF7L2 effect seems to be much higher than any other confirmed T2D candidate to date (Cauchi *et al.*, 2007). Studies involving non-European ethnic backgrounds, including Indian (Chandak *et al.*, 2007), Japanese (Hayashi *et al.*, 2007) and Moroccan populations (Cauchi *et al.*, 2007), also indicated positive association between TCF7L2 variants and T2D. This was also reported in Indian-Asian (Humphries *et al.*, 2006; Bodhini *et al.*, 2007) and Pakistani (Rees *et al.*, 2008) populations. More recently, a lack of association between a TCF7L2 rs7903146 variant and type 2 diabetes was reported in Arabian Emirates (Saadi *et al.*, 2008), as well as in an Arab population of Saudi origin (Alsmadi *et al.*, 2008), although this was completely inconsistent with previous reports. The aim, hereby, was to investigate the association between the TCF7L2 rs7903146 variant and T2D in an Iranian population.

The study group comprised 258 type 2 diabetes patients, recruited from a diabetes clinic in the Aliebn Abitaleb hospital, Rafsanjan University, southeast Iran. Patients with diabetes were selected based on the following criteria: ≥ 126 mg/dL on two different occasions and abstinence from insulin treatment. Patients presenting anti-insulin or anti-GAD antibodies were ruled out. 168 normal healthy control subjects were recruited from the same area, and were age-matched with the case population. In these, the absence of T2DM was certified by an expert endocrinologist, based on the above criteria. All the subjects selected for this study were of Fars origin. Population admixture is rare in this area and subjects with other ethnic backgrounds were not included in the study.

Following registration and the provision of personal and demographic data, 3-5 cc of venous blood was collected in EDTA tubes and stored at -20 C for DNA extraction. The study was approved by the Ethics committee of Tehran University. Informed consent was obtained from each patient involved. DNA was extracted by the salting-out method from anti-coagulated blood collected in EDTA tubes. ARMS- PCR (Amplification Refractory Mutation System) was employed for genotyping. The following primers were used:

Common forward primer: 5' - ggagc cgtca gatgg taatg -3'

TCF4-C: 5' - ggtg cctca tacgg caatt aaatt atatag -3'

TCF4-T: 5' - ggtg cctca tacgg caatt aaatt atataa -3'

β-actin- F: 5' - ctt cct tcc tgg gca tgg ag -3'

β-actin- R: 5' - tgg agg ggc cgg act cgt ca -3'

The PCR cycles were as follows: 5 min at 95 °C, followed by 35 cycles of 30 s at 95°, 30 s at 66 °C and 60 s at 72 °C. Final extension was 7 min at 72 °C. The presence of PCR product (188 bp for TCF7L2 and 315 bp for β-actin) was verified on 2% agarose gel stained with ethidium bromide. Genotyping results were validated by direct sequencing.

Odd ratios (OR) and 95% confidence intervals (CI) were used for estimating the strength of association between different groups and alleles or genotypes of TCF7L2 gene polymorphism. Significance levels were defined through either Chi-quare or Fisher exact analyses with contingency tables. All analyses were carried out using STATA (v8) software.

Genotype frequencies did not differ from the expected Hardy-Weinberg ratios. The Male/Female ratio was 68/170 in diabetic patients and 58/72 in controls. The mean age was 53 ± 10 in diabetic patients and 52 ± 10 in normal controls. The mean BMI was 28 ± 4 in patients with diabetes and 26 ± 3 in normal controls.

Allele and genotype frequencies for TCF7L2 rs7903146 variation was significantly different in cases compared to the controls TT *vs.* CT +CC [p 0.0081 OR 3.4 95%CI(1.27-11.9)] and T *vs.* C allele [p 0.02 OR 1.4 95%CI (1.03-1.9)] Table 1.

It was found that the T allele of TCF7L2 rs7903146 is associated with T2D in the Iranian population. TCF7L2 is a high mobility group (HMG) box-containing transcription factor which is part of the Wnt signaling pathway (Grant *et al.*, 2006). The expression of this gene is also associated with insulin secretion in human pancreatic β cells both *in vivo* and *in vitro* (Lyssenko *et al.*, 2007)

In most recently published studies, it has been suggested that TCF7L2 is associated with impaired insulin secretion, but not with increased insulin resistance (Florez *et al.*, 2006; Loos *et al.*, 2007; Lyssenko *et al.*, 2007). Also a multiplicative interaction between this variant and obesity or high BMI was inferred from two previous studies (Yan *et al.*, 2008). Both imply that the risk of type 2 diabetes is greater in lean individuals carrying this polymorphism, whereas no significant association was noted in those obese or overweight. Therefore it seems that the SNP rs7903146 is a much more influential risk factor for lean individuals than for obese (Yan *et al.*, 2008). Helgason *et al.* (2007) reported that the rs7903146 T allele, probably an ancestral and not causative variant, tags an unidentified functional variant lying outside the screened locus.

Our data confirm the association of this widely replicated variant with T2D risk in Iranians, in contrast with previous reports in Arab populations. Differences in ethnic background or the effect of environmental factors, such as life-style, may explain these discrepancies.

## Figures and Tables

**Table 1:** TCF7L2 rs7903146 polymorphism allele and genotype frequencies in patients with type2 diabetes and controls.

	Diabetes	Controls			
No.patients	(N = 258)	(N = 168)	p value	OR	95% CI
Genotype					
CC	95 (37%)	76 (45%)			
CT	138 (53%)	87 (52%)			
TT	25 (10%)	5 (3%)	0.0081	3.4	(1.27-11.9)
Allele (2N)^2^					
C	328 (64%)	239(71%)	0.02	1.4	(1.03-1.9)
T	188 (36%)	97 (29%)			
